# Membranous nephropathy

**DOI:** 10.1590/2175-8239-JBN-2023-0046en

**Published:** 2023-07-31

**Authors:** Márcio Dantas, Lázaro Bruno Borges Silva, Barbhara Thaís Maciel Pontes, Marlene Antônia dos Reis, Patrícia Soares Nunes de Lima, Miguel Moysés

**Affiliations:** 1 Universidade de São Paulo, Faculdade de Medicina, Hospital das Clínicas, Ribeirão Preto, SP, Brazil.; 2 Universidade Federal do Triângulo Mineiro, Patologia Geral, Centro de Pesquisa em Rim, Uberaba, MG, Brazil.

**Keywords:** Glomerulonephritis, Membranous, Autoantibodies, Rituximab, Cyclophosphamide, Steroids, Calcineurin inhibitors, Glomerulonefrite membranosa, Autoanticorpos, Rituximabe, Ciclofosfamida, Corticosteroides, Inibidores de Calcineurina

## Abstract

Membranous nephropathy is a glomerulopathy, which main affected target is the podocyte, and has consequences on the glomerular basement membrane. It is more common in adults, especially over 50 years of age. The clinical presentation is nephrotic syndrome, but many cases can evolve with asymptomatic non-nephrotic proteinuria. The mechanism consists of the deposition of immune complexes in the subepithelial space of the glomerular capillary loop with subsequent activation of the complement system. Great advances in the identification of potential target antigens have occurred in the last twenty years, and the main one is the protein “M-type phospholipase-A2 receptor” (PLA2R) with the circulating anti-PLA2R antibody, which makes it possible to evaluate the activity and prognosis of this nephropathy. This route of injury corresponds to approximately 70% to 80% of cases of membranous nephropathy characterized as primary. In the last 10 years, several other potential target antigens have been identified. This review proposes to present clinical, etiopathogenic and therapeutic aspects of membranous nephropathy in a didactic manner, including cases that occur during kidney transplantation.

## RESUMO

A nefropatia membranosa é uma glomerulopatia, cujo principal alvo acometido é o podócito, e acarreta consequências na membrana basal glomerular. Tem maior frequência em adultos, principalmente acima dos 50 anos. A apresentação clínica é a síndrome nefrótica, mas muitos casos podem evoluir com proteinúria não nefrótica assintomática. O mecanismo consiste na deposição de complexos imunes no espaço subepitelial da alça capilar glomerular com subsequente ativação do sistema do complemento. Grandes avanços na identificação de potenciais antígenos alvo têm ocorrido nos últimos vinte anos, e o principal é a proteína “M-type phospholipase-A2 receptor” (PLA2R) com o anticorpo anti-PLA2R circulante, o que possibilita avaliar a atividade e o prognóstico dessa nefropatia. Essa via de lesão corresponde aproximadamente a 70% a 80% dos casos da nefropatia membranosa caracterizada como primária. Nos últimos 10 anos vários outros antígenos alvo potenciais têm sido identificados. Esta revisão se propõe a apresentar de modo didático aspectos clínicos, etiopatogênicos e terapêuticos da nefropatia membranosa, incluídos os casos com ocorrência no transplante renal.

## Introduction

Membranous nephropathy (MN) is a glomerulopathy defined by very characteristic morphological findings that include subepithelial immune deposits in the glomerular capillary loops. The clinical picture consists of nephrotic syndrome (NS) or asymptomatic proteinuria and, although it may occur in any age group, it is rare in children and predominates in adults over 50 years of age. In the last two decades, potential target antigens have been identified. The main antigen is the “M-type phospholipase-A2 receptor” (PLA2R), described in 2009. The serum dosage of the anti-PLA2R antibody has considerably modified criteria such as clinical and immunological activity or remission, in addition to serving as a prognostic parameter and indication of immunosuppressive treatment. Since 2014, other target antigens have also been discovered (THSD7A, EXT1/2, NELL1, Sema3B, NCAM1, PCDH7, HTRA1 and NTNG1). Some of these antigens have shown associations with membranous nephropathy with some features, such as, for example, Sema3B predominating in children, THSD7A in some neoplasms, EXT1/2 with systemic lupus erythematosus and other systemic autoimmune diseases. A change in classification has also been suggested based on the association with the respective antigen involved.

Despite these advances, the lack of knowledge of triggers for the onset of the disease, the participation of different subclasses of IgG (IgG1, IgG2, IgG3 and IgG4), the complement system pathways involved, and the participation of other mediators of the immune system, such as changes in of regulatory T cells, have hindered a more comprehensive understanding of disease mechanisms. In addition, the available therapeutic options have relatively low remission rates and high adverse events.

This review aims to present the clinical characteristics in a didactic way, highlighting the etiopathogenic mechanisms and therapeutic regimens recommended by international NM guidelines, including cases that occur in kidney transplantation.

## Epidemiology

MN is the main cause of nephrotic syndrome in non-diabetic white adults (about 30%), with an estimated annual incidence of 10–12 cases per million/year in the North American population^1,2^. In Brazil, considering primary glomerulopathies, MN is the second most frequent diagnosis in native kidney biopsies (20.9%). However, biopsy indications, genetics and environmental characteristics may influence the epidemiology of glomerulopathies^3,4^. Patients of all age groups can develop MN, with a median age of 50–60 years and a higher prevalence in men (2:1)^2^. About 20% of patients are older than 60 years at the time of diagnosis. Involvement in children is rare. Primary MN associated with anti-PLA2R antibody typically affects men (75% of cases), at a median age of 52 years. In contrast, MN associated with systemic autoimmune disease occurs more frequently in women (81% of cases) at a young age. MN associated with malignancy affects older patients, with a median age of 65 years^5^.

## Clinical and Diagnostic Framework

**Figure 1.**
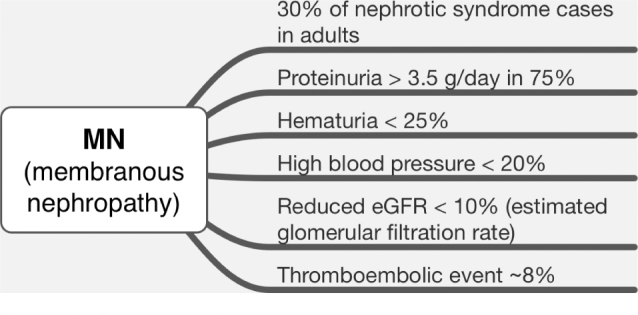
Frequency of clinical features at the presentation of the membranous nephropathy.

The clinical presentation of MN is heterogeneous, but most cases (70–80%) present insidiously and with high 24-hour proteinuria (>3.5 g/24h), associated with peripheral edema or anasarca, hypoalbuminemia (<2g/dL) and lipuria. Presentation with non-nephrotic proteinuria (<3.5 g/24h) is less frequent. However, in these cases, there is an increase in proteinuria to nephrotic levels in up to 60% of cases during the first year of follow-up^6^. The frequency of clinical manifestations in the presentation of MN is shown in Figure 1.

Other findings may be found less frequently, such as microscopic hematuria (25–50%), arterial hypertension (20–50%) and changes in renal function (25%)^2,7^. These alterations raise the suspicion of secondary MN or of some complication of the disease, such as acute kidney injury or evolution with already present chronic nephropathy. MN accompanied by hematuria with reduced glomerular filtration and newly installed arterial hypertension may be indicative of concomitant mesangial hypercellularity, which occurs not only, but mainly, in class V lupus nephritis. Other forms of secondary MN may present with this same presentation.

In the presence of hypoalbuminemia, there is a high risk of thromboembolic events due to the imbalance of factors in the coagulation cascade, especially renal vein thrombosis; as well as dyslipidemia, with increased levels of LDL and VLDL fractions of serum cholesterol, secondary to lipoprotein lipase deficiency^8^. Serum levels of C3, C4 and CH50 are normal, despite the renal presence of complement components.

The clinical evolution of MN cases is also heterogeneous and may present spontaneous total remission (20%-30% in five years and especially if proteinuria is less than 8 g/day), partial remission (20%–25% in 5 years), gradual evolution to end-stage chronic kidney disease (40%–50% in 10 years) or rapidly progressive acute kidney injury^1,2,7^. Follow-up for 5 years is required to determine the complete remission rate. MN relapses usually occur in cases of partial remission and when immunosuppressive treatment is discontinued^1^. Some factors are directly related to prognosis, such as age over 50 years, intensity and evolution of proteinuria, high serum creatinine, presence of glomerular sclerosis and interstitial fibrosis, and tubular atrophy.

Renal biopsy is the reference method for establishing the diagnosis of MN. However, in selected situations, it can be dispensed with^9^. According to recommendations from KDIGO 2021 (Kidney Disease: Improving Global Outcomes)^10^, in patients with nephrotic syndrome and stable GFR, the serum dosage of anti-PLA2R antibody by ELISA and indirect immunofluorescence assay may be sufficient. If the anti-PLA2R antibody test is negative, or if this assessment is not feasible, a renal biopsy should be performed^9^.

In certain cases, even in the presence of anti-PLA2R antibody, biopsy is indicated as it provides additional and potentially relevant information to the diagnosis and prognostic evaluation: (a) atypical clinical course, especially if there is a rapid decline in glomerular filtration; (b) laboratory alteration not compatible with MN associated with PLA2R, in particular autoimmune markers, such as positive antinuclear antibody, and (c) unsatisfactory response to immunosuppressive treatment with progressive worsening of glomerular filtration, or even in the persistence of nephrotic syndrome after the disappearance of anti-PLA2R.

When an underlying systemic etiology (autoimmune, neoplastic or infectious) is not identified, MN is considered primary and can be understood as an autoimmune disease limited to the kidney. Concomitant with the identification of certain podocyte target antigens of immune aggression in MN, the old “idiopathic” terminology began to be gradually abandoned. The classification of MN in primary or secondary forms has several limitations, which is why new classification proposals have been presented, such as a molecular classification that associates MN to the respective antigen, for example, MN associated with PLA2R5.

**Figure 2.**
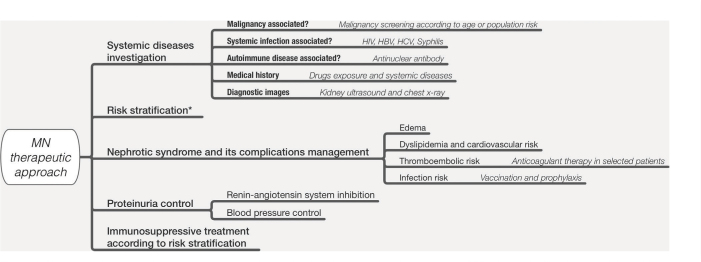
Algorithm with suggestions to approach according to the risk of progression. Abbreviations: GFR: glomerular filtration rate. Adapted from Alsharhan and Beck Jr^1^.

As recommended by KDIGO 2021^10^, some more frequent systemic diseases that have the potential to cause MN, such as hepatitis B and C, HIV, syphilis and systemic lupus erythematosus (SLE), should be investigated routinely in the clinical presentation. Neoplastic diseases should also be actively investigated when there is weight loss, anemia or family or environmental history. Figure 2 presents an algorithm for a treatment plan that considers the most frequent causes of secondary MN. The identification of systemic disease compatible with secondary MN directs the approach to the specific etiology. In these cases, remission of the nephropathy is expected with treatment of the underlying disease.

## Etiopathogenic Mechanisms

Knowledge of the mechanisms involved in MN increased as of 1959, when the experimental model of Heymann’s nephritis was developed in rats^11^. In this experimental model, the target antigen was LRP-2/megalin, present in rats in the brush border of proximal tubule cells and also in the foot processes of podocytes^12^. Among the most relevant findings with this model are the demonstration of “in situ” formation of the immune complex in the subepithelial space and the need for complement system activation for the development of proteinuria^13,14^. However, this antigen does not exist on podocytes in humans, and thus, for about 40 years, human MN remained without the identification of any target antigen.

### Target Antigens Identification in Humans

Knowledge of target antigens in humans began in 2002 with the identification of the neutral endopeptidase protein (NEP), involved with a rare type of MN^15^. In this case, a newborn with nephrotic syndrome due to MN, confirmed by renal biopsy days after birth, had immune deposits containing IgG and C3, located by electron microscopy in the subepithelial space. As a triggering mechanism, the mother was genetically deficient for the NEP protein. The pregnant woman had been alloimmunized in a previous pregnancy and in the following pregnancy there was placental transfer of maternal anti-NEP antibodies to the fetus.

However, the NEP antigen is not involved in the vast majority of cases of MN. After seven years, the protein “M-type phospholipase-A2 receptor” (PLA2R) was identified as a target antigen^16^, in this case as an autoantigen, unlike the MN induced by alloimmunization and placental transfer previously described. PLA2R is a transmembrane glycoprotein widely expressed in human podocytes both in the podocyte processes and on the apical surface with little known function. The trigger for the production of anti-PLA2R antibodies remain unknown. Anti-PLA2R antibodies were found in the serum of 70%–80% of patients with primary MN. It is noteworthy that the anti-PLA2R antibody is associated with primary MN but has also been identified in the replication process in hepatitis B virus infection^17,18^ and in cases of Sarcoidosis^19^. The participation of the anti-PLA2R antibody in the pathogenesis of MN was reinforced in an experimental study with a strain of pigs that express PLA2R in the kidney. These animals developed proteinuria after administration of plasma or purified antibody from patients with PLA2R20-associated MN.

It is also worth mentioning that the anti-PLA2R antibody can be measured in the blood using the ELISA method, with a specificity of 99.6%, and through an indirect immunofluorescence assay (does not allow quantification), being 100% specific, through commercial kits (both Euroimmun®).

After five years, another podocyte target antigen named “thrombospondin type 1 domain-containing 7A (THSD7A) was identified^21^. This antigen frequently occurs in 1% to 3% of PLA2R-negative MN cases. It is worth mentioning that the cases of NM associated with anti-THSD7A antibodies were related to some neoplasms. Furthermore, successful antineoplastic treatments have induced remission of the nephrotic syndrome and this protein has already been identified in some types of neoplastic cells^22^.

Until then, human antigens were identified through methods based on “western-blotting” using patient sera and solubilized normal glomerular extracts. The band obtained on the gel was removed and the antigen identified by mass spectrometry. Since 2019, a new research approach for glomerular target antigens has been used. From the renal biopsy tissue, the glomeruli of patients with MN were cut and isolated by laser microdissection followed by the identification of target antigens by mass spectrometry^23^.

The first antigens identified with this new approach were exostosins 1 and 2 (EXT1/2)^23^. Anti-EXT1/2 antibodies are present in subepithelial deposits and are associated with SLE (about 30%) and with other autoimmune diseases. About 80% of the patients with the anti-EXT1/2 antibody in the biopsies were women (mean age: 35 years); and about 70% of the patients had serum alterations of autoimmune diseases. Most of these patients also had renal biopsy with signs suggestive of secondary MN such as C1q deposits; IgG1 as the predominant immunoglobulin, mesangial and subendothelial immune deposits, and tubuloreticular inclusions in endothelial cells^23^. However, there are no reports of serum anti-EXT1/2 antibody, which makes it difficult to characterize it as a well-established target antigen.

Other potential target antigens have been identified using this same technique. The NELL-1 protein (“neural epidermal growth factor-like 1 protein”) was described in 2020, and identified in about 20% of PLA2R negative patients, and the anti-NELL-1 antibody was identified in the serum of patients^24^. The immune deposits of these patients contained all IgG isoforms, with IgG1 being the most intense and IgG4 being the least intense. These patients also showed a greater association with the occurrence of neoplasms^24,25^.

Another potential target antigen is the NCAM1 protein (Neural cell adhesion molecule 1), and its identification occurred in 2021 from frozen kidney samples^26^. Anti-NCAM1 serum antibodies were identified in about 6% of patients with lupus MN, which classifies this histopathological marker as another potential target antigen of this nephropathy. This opinion is reinforced by the fact that the clinical and histopathological alterations were similar to those in the study for EXT1/2. However, this and antibody was also seen less frequently in patients with primary MN.

Semaphorin 3B (Sema 3B), also described in 2020^27^, predominated in pediatric patients, although it was also identified in adults. Anti-Sema3B antibodies were found in the patients’ serum and were associated with clinical and histopathological features suggestive of secondary MN and no IgG4 deposition. So far, this target antigen is the pioneer among cases of pediatric idiopathic MN.

More recently, “protocadherin 7” (PCDH7) was identified in 14 cases with the presence of circulating antibodies and with evidence of secondary MN^28^; “serine protease high-temperature requirement A1” (HTRA1), which also occurred in 14 cases and with circulating anti-HTRA1 antibodies, and also with signs of secondary disease^29^, and “Netrin G1” (NTNG1), which was seen in only 3 patients, but without the detection of circulating antibodies^30^.

There is a plausible hypothesis that, in cases of MN classified as secondary^1^, the immune complex formation begins with the generation of neoantigens or by antigens “planted” in the subepithelial space followed by the subsequent binding of the respective antibody^31^. This hypothesis is reinforced by studies that detected antigens in the subepithelial space of the glomerular capillary loop in cases of secondary MN, which may explain the pathophysiology of these nephropathies, as in cases with human thyroglobulin antigen^32^, with antigen “e” of hepatitis B33, and with the cationic bovine serum albumin as ingested exogenous antigen^34^.

### Who may be at Risk to Develop MN?

We have known for some decades now that some alleles of the HLA class II system, such as HLA-DR3 and HLA-DQA1, show a strong association with MN^35,36^. An association has also been reported between single nucleotide variations (SNVs) of the HLA class II HLA-DQA1 complex gene (SNP rs2187668) from chromosome 6p21 and the PLA(2)R1 receptor gene (SNP rs4664308) from chromosome 2q24 in French, German and English populations with MN^37,38^.

In the sequence of immunological events, we know how the loss of tolerance to self-antigens occurs, but some studies have shown dysfunction of B and T cells with proportional reduction of regulatory T cells^39,40,41^.

### How Does Podocyte Injury Occur After Immune Complex Deposition?

The need for complement system activation with formation of the membrane attack complex (C5b-9 components) was demonstrated in the experimental model of Heymann’s nephritis^42^. The formation of the membrane attack complex generates sublethal damage to the podocyte with disruption of the actin cytoskeleton, loss of the glomerular cleft diaphragm and cell dysfunction, which results in proteinuria and production of glomerular basement membrane with altered composition^43^. However, many questions remain about how and which complement pathways are involved. Primary MN has a predominance of the IgG4 subclass, which does not activate the complement system. More recent studies have suggested that deposits in the initial phases have a greater amount of IgG1 and IgG3, which would activate the classic complement pathway, while deposits in the more advanced phases would have a greater amount of IgG4, suggesting a more pronounced participation in the alternative lectin pathway^44,45,46,47^. A detailed understanding of these mechanisms is important for the development of new treatments, such as the use of complement system activity attenuators.

## Morphology

The anatomopathological diagnosis of MN is defined by the deposition of immune deposits in a subepithelial location in the glomerular capillary loop. It also encompasses the spectrum of changes in the glomerular basement membrane as a result of aggression mediated by immune deposits. Kidney biopsy also has prognostic relevance by identifying active, potentially reversible lesions and/or chronic lesions, such as interstitial fibrosis, tubular atrophy and glomerular sclerosis^6,48^.

The understanding of glomerular morphological changes in patients with nephrotic syndrome was boosted by the development of histological techniques in the late 1940s, mainly including immunohistochemistry. The first description of the morphological pattern characterized by the thickened aspect of the basement membrane of the glomerular capillary loops in adult patients with nephrotic syndrome dates from this period^49^.

This pattern of glomerular injury, called “membranous”, was perfected by Jones^50^ in 1957 through the methenamine silver impregnation technique. Reported changes have included thickening of the basement membrane of glomerular capillaries, irregular protrusions of the mesangial matrix with an irregular, silver-positive, spike-like appearance; in some patients, where the lesion occurred later, there were alterations of the lamellation type and formation of chain-link lesions.

**Figure 3.**
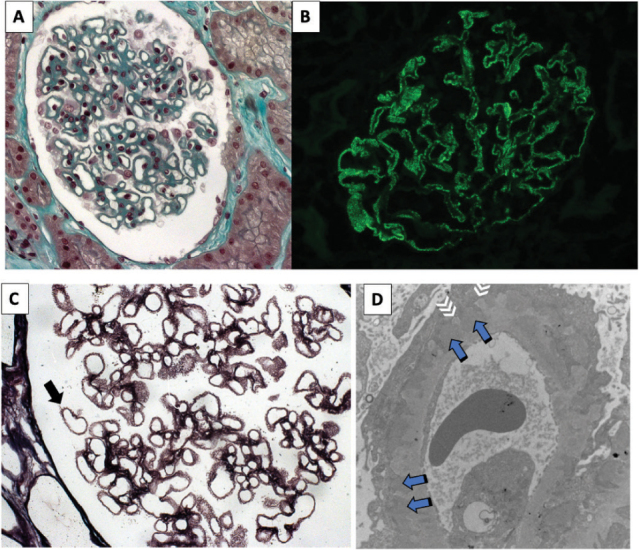
Morphologic findings in the membranous nephropathy. A: glomerulus with global thickening of the capillary wall. (light microscopy, Masson trichrome, 40×). B: positive, high-intensity granular, in glomerular walls (immunofluorescence microscopy, 40×). C: capillary walls with spikes at the basement membrane in stage 2 membranous nephropathy (light microscopy, silver methenamine staining, 100×). D: electron-dense deposits and thickening of the basement membrane with spikes at the subepithelial aspect of the glomerular capillary wall; blue arrows: subepithelial electron-dense deposits at the subepithelial aspects of the glomerular basement membrane; White arrows: basement membrane projections enveloping the deposits; (electron microscopy, 7000×). A, B and C courtesy of Prof. Roberto Silva Costa (Ribeirão Preto Medical School, University of São Paulo, Brazil).

The immune deposits located between the podocyte and the glomerular basement membrane are composed of the podocyte target antigen (PLA2R, SEMA-3B, THSD7A, among others), an immunoglobulin G (Figure 3B), usually with a predominance of the IgG4 subtype (Figure 3C), mainly in PLA2R-associated MN, and by complement fractions. In the early stages of the disease, when there is no thickening of the capillary loop visible on light microscopy (LM), changes are identified only through immunofluorescence (IF) and electron microscopy (EM) techniques^1,51^.

By immunofluorescence, the subepithelial immune deposits of IgG and C3 result in the typical global and diffuse finely granular pattern in glomerular capillary loops (Figure 3B). Complementary evaluation with immunohistochemistry can reveal IgG subclasses (which is not routinely performed) or mark the podocyte antigen associated with the immune deposit. Through the EM, the deposits show an electron-dense appearance and subepithelial location (Figure 3D).

When the aggression mediated by subepithelial immune deposits is triggered, the subsequent alterations of the epithelial cell and basement membrane can be recognized in the different evolutionary stages of the disease using LM, EM, immunohistochemistry and IF. There is podocyte injury with simplification, with enlargement of the podocyte pedicels, and loss of the slit diaphragm; as the podocyte continues to produce its basement membrane (“turnover”), this material is initially located between one immune deposit and another (Figure 3C and 3D), and then on the immune deposits, surrounding them; finally, overall thickening of the capillary loop occurs (Figure 3A)^52^.

### Morphological Classification

The sequence of histopathological alterations initiated from the immune deposition is presented in the morphological classification (Table 1) proposed by Ehrenreich and Churgh^53^ in 1968. This classification describes 4 sequential stages, characterized by the predominant morphological aspect of the base membrane and the immune deposits (initially electron dense, when in stage 1; or electron-lucency later, in stage 4, when they tend to be reabsorbed and incorporated into the basement membrane).

**Table 1.** Histopathology Changes Caused by Membranous Nephropathy

Microscopy → Stage ↓	LM	IF	EM
**Stage 1**	Normal GCL	IgG fine granular in GCL	Subepithelial electron-dense deposits
**Stage 2**	Thick GCL with GBM spikes (MSS)	IgG granular in GCL	Subepithelial electron-dense deposits with spikes
**Stage 3**	Thick GCL and with chain links (MSS)	IgG granular in GCL	Subepithelial electron-dense deposits involved by the GBM
**Stage 4**	Thick GCL with variable changes (MSS)	IgG granular and GCL variable	GBM with variable irregularities

LM: light microscopy; IF: immunofluorescence microscopy; EM: electron microscopy; GCL: glomerular capillary loop; MSS: methenamine silver stain; GBM: glomerular basement membrane.

The first of these (MN stage I) represents the initial period of glomerular injury; subepithelial and electron dense deposits are small, often sparse, which is why they have a fine granular appearance when identified by IF. Changes in GBM, as a rule, are subtle, or even absent; there is no thickening or projections, although discrete membrane depressions can be noted in some cases.

In subsequent stages, as the basement membrane is continuously produced, irregularities, thickening and projections of the GBM are noted that can be inserted between the immune deposits (called GBM spicules), characteristic of stage II, or involve them completely (aspect in “chain link”), which characterizes stage III. In the latter, the resulting appearance of deposits surrounded by this new basement membrane (“neo membrane”) may give an intramembranous appearance to GBM. These basement membrane changes are seen under methenamine silver (MS) impregnation and/or under EM.

Electrodense deposits, numerous and more voluminous than deposits in stage I, result in the granular, global and diffuse pattern revealed in strong intensity by immunofluorescence (stages II and III). In contrast, in stage IV, the deposits lose their electron-dense appearance as they are incorporated into the basement membrane. At this stage, GBM may show variable irregularities when observed by LM and/or EM.

Finally, it is important to note that, although this classification describes the morphological changes in their probable evolutionary sequences, its correlation with the clinical course of the disease (proteinuria, worsening of renal function and progression to chronic kidney disease) is uncertain.

### Morphological and Etiopathogenic Correlation

Histological alterations help to identify secondary forms of MN. Mesangial and/or endocapillary hypercellularity; mesangial matrix expansion; leukocyte infiltration; and, sometimes, cell crescents are suggestive of glomerulopathy secondary to a neoplastic, autoimmune or infectious systemic process. The clinical repercussions of these lesions, not infrequently, include hematuria, arterial hypertension and changes in glomerular filtration, which are uncommon in the primary forms.

Strong immunofluorescence positivity with antisera other than IgG and C3 is also suggestive of secondary MN and may recommend an active search for neoplasms, infections, and autoimmune diseases. Among the secondary forms, class V of lupus nephritis is relevant due to its higher frequency in clinical practice. In these cases, the search for the EXT1/EXT2 antigen, when available, can be performed by immunohistochemistry, and its positivity in a fine granular pattern suggests class V lupus nephritis, or MN secondary to another systemic autoimmune disease^46^. The identification of EXT1/EXT2 as a target antigen in class V MN of lupus nephritis apparently results in a better prognosis^54^.

**Figure 4.**
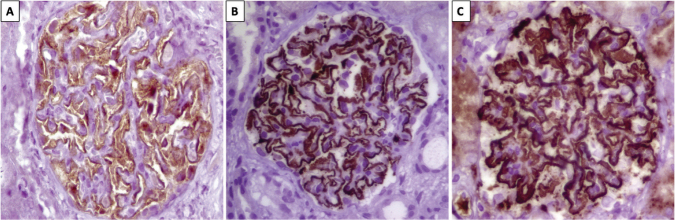
PLA2R Immunohistochemistry. A. Control: negative PLA2R. No granular immunostaining at the capillary walls; there is normal scattered PLA2R immunostaining in podocytes. B: positive granular global PLA2R immunostaining at the basement membrane of the glomerular capillary walls; C: Positive IgG4. Granular immunostaining at the basement membrane of the capillary walls (40×). Primary antibodies: anti-PLA2R (1:2500, Sigma) and IgG4 (1:3000, Gene Tex).

Identification of the predominant IgG subtype can be useful in distinguishing between primary and secondary forms of MN; however, it has limited value when used alone. The IgG4 subclass (Figure 4C) is the predominant subtype in primary forms of MN (as in PLA2R-associated MN, Figure 4B), while in secondary forms (autoimmune or neoplastic), IgG1, IgG2 or IgG3 can be predominant^46^.

The changes seen by light, immunofluorescence and electron microscopy are shown in Figure 3A-D. The IgG4 subclass is exemplified by immunohistochemistry in Figure 4C. The presence of PLA2R as a target antigen in the glomerular capillary loop in a case of primary MN is demonstrated in Figure 4B.

## Approach and Treatment

The treatment of patients with MN must be individualized. Among the considerations that precede the definition of the treatment plan, there is the distinction between primary or secondary disease, with the identification of the podocyte antigen involved when possible; the stratification of the kidney disease risk of progression or the possibility of spontaneous remission; and, finally, the treatment of the nephrotic syndrome itself with its potential complications (edema, thrombotic events, infections)^1,2,10,55^. Figure 2 presents a treatment plan for MN based on: investigation of systemic diseases as causes of secondary MN; risk stratification; management of complications of nephrotic syndrome; conservative treatment to reduce proteinuria and nephroprotection, and immunosuppressive treatment.

Treatment with supportive measures should be established for all patients diagnosed with MN, with emphasis on blood pressure control; diet adequacy with reduced sodium intake; proteinuria reduction by blocking the renin-angiotensin system; dyslipidemia control, and risk of thromboembolic event assessment with decision on prophylactic anticoagulation in nephrotic syndrome with severe hypoalbuminemia, particularly in those with serum albumin <2.5g/dL^10^ (Figure 2).

**Figure 5.**
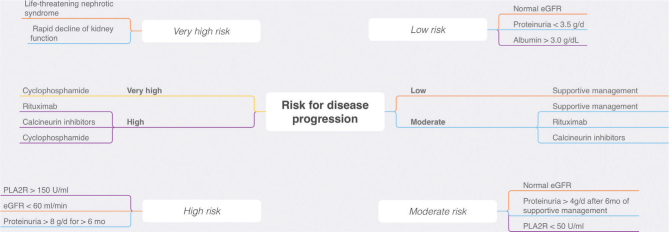
Diagnostic and therapeutic approach to the patient with membranous nephropathy. HIV: human immunodeficiency virus; HBV: hepatitis B virus; HCV: hepatitis C virus.

Figure 5 presents treatment recommendations based on the risk of MN progression: low, moderate, high and very high. Knowledge of the natural history of MN brought valuable information with great applicability in clinical practice. Up to 30% of patients with MN may experience spontaneous remission of proteinuria, with good long-term renal prognosis (low risk of progression to end-stage chronic kidney disease). In these cases, immunosuppression is not necessary and the treatment of choice is supportive therapy. Stratifying the risk of kidney disease progression; therefore, it is essential to identify patients who can potentially benefit from immunosuppressive therapy. Immunosuppressive treatment can be postponed for 3 to 6 months in cases with risk characterized as low or moderate, because there is a chance of spontaneous remission. However, in the most severe cases, immunosuppression should be instituted soon after the diagnosis^1,2,10,55^.

Following the recommendations of KDIGO and other reviews, immunosuppression is not necessary in patients with proteinuria <3.5 g/24h and estimation of glomerular filtration (eGFR) > 60 ml/min/1.73m^21,2,10,55^. In cases stratified as moderate risk, when there is no potentially serious complication of nephrotic syndrome (thrombotic event, infection or acute kidney injury) and glomerular filtration is normal, conservative treatment can be tried for 3–6 months before starting immunosuppression.

For risk progression stratification, in addition to proteinuria and eGFR, measurement of serum anti-PLA2R antibody has been incorporated into clinical practice^1,2,10^ (Figure 5). When available, it provides prognostic information and correlates with disease activity. Low serum titers (PLA2R Ab < 50 RU [reference units]/mL) are associated with a greater likelihood of spontaneous remission, whereas high titers (PLA2R Ab > 150 RU/mL) are indicative of a high risk of progression. PLA2R Ab serum titers < 14 RU/mL are considered normal, and titers < 2 RU/mL characterize complete immunological remission^10^. It is worth mentioning that these reference values are not fully validated and the risk stratification must consider other criteria.

Immunosuppressive treatment should be initiated in cases with: (a) reduction in glomerular filtration (eGFR < 60 mL/min/1.73 m^2^) associated with MN (without another pertinent justification for the change in GFR); b) severe nephrotic syndrome (acute kidney injury, thrombotic event or infection), and c) in nephrotic patients who do not respond satisfactorily to conservative treatment.

The choice of immunosuppressive regimen will depend on the risk stratification and patient characteristics. It is important to highlight that steroid monotherapy is ineffective and it is not indicated in MN.

The clinical response, whatever the immunosuppressive regimen used, must be evaluated during the course of treatment. Definitions of clinical response include complete remission, characterized by reduction in proteinuria to values below 0.3 g/day and normalization of serum albumin; partial remission, characterized by proteinuria < 3.5 g/day with a minimum reduction of 50% from baseline and eGFR stabilization; relapse, characterized by recurrence of proteinuria > 3.5 g/day after remission, and therapeutic failure to maintain levels > 3.5 g/day and absence of a minimum reduction of 50% in baseline proteinuria.

### Cyclophosphamide

One study demonstrated a higher rate of complete remission and renal survival with the combined use of chlorambucil and steroid (6 months of treatment) compared to the control group. Subsequently, the original regimen with chlorambucil was compared to the use of cyclophosphamide associated with steroid, resulting in similar outcomes^56,57^.

The scheme with oral cyclophosphamide associated with steroid, called “modified Ponticelli”, is used as the preferred therapy in very high-risk patients, that is, when there is a rapid decline in renal function and in severe nephrotic syndrome (with a life-threatening event, as in cases associated with a severe thrombotic event).

The main side effects associated with cyclophosphamide are: infertility, increased susceptibility to infections, increased risk of malignancy (especially with a cumulative level greater than 36 grams), bladder cancer and myelodysplasia. It is important to regularly evaluate the CBC due to the risk of anemia, leukopenia and thrombocytopenia. The use of trimethoprim-sulfamethoxazole for Pneumocystis carinii prophylaxis should be considered during immunosuppression with cyclophosphamide.

Medication and dosage (modified Ponticelli scheme):

- Months 1, 3, and 5: Methylprednisolone 1g (IV) for 3 days, followed by prednisone 0.5 mg/kg/day (PO) for 27 days.
- Months 2, 4 and 6: Cyclophosphamide 2.0–2.5 mg/kg/day (PO).

### Calcineurin Inhibitors

A sound treatment option in cases of moderate or high risk and for diabetic patients. It is also the therapy of choice for patients of childbearing age. Low-dose prednisone should be associated with a calcineurin inhibitor. During treatment with cyclosporine, reduction in proteinuria may be slow. Treatment failure may be considered after 6 months if there has been no reduction in proteinuria. Recurrence may occur after withdrawal of medication. In these cases, the medication can be reintroduced, or the regimen can be changed. As adverse events, the nephrotoxicity of cyclosporine and tacrolimus stands out. Furthermore, cyclosporine can cause hypertrichosis and gingival hypertrophy; tacrolimus can cause seizures, among other adverse events.

Medication and dosage:

- Ciclosporin: 3.5–5.0 mg/kg/day in two doses; recommended serum level (valley dosing): 120–200 µg/L; duration: 12–18 months;

Or:

- Tacrolimus: 0.05–0.075 mg/kg/day in two doses; desired serum level: 3–5 µg/L; duration: 12–18 months;

### Rituximab

Rituximab is an anti-CD20 monoclonal antibody currently considered the therapy of choice in refractory disease, in addition to being an option as initial therapy in cases of moderate or high risk. The Membranous Nephropathy Trial of Rituximab (MENTOR) study compared the use of cyclosporine at a dose of 3.5–5 mg/kg/day for 6 months with rituximab (1 g/dose, with an additional dose of 1 g after 2 weeks. In this study, there was no inferiority of rituximab in relation to the use of cyclosporine (sustained remission rates after 12 and 24 months). As to adverse events, there may be infusion-related reactions (rash or anaphylaxis in more severe cases). Pre-treatment with dexamethasone and diphenhydramine should be performed and may reduce the risk of these reactions. The risk of infections during treatment with rituximab is associated with further B-lymphocytes depletion. There is therefore a risk of hepatitis B and tuberculosis reactivation. Previous treatment at the beginning of immunosuppression is indicated for patients with latent infection or previous exposure to these infections^58^.

Posology:

- 375 mg/m2/week intravenously for 4 weeks;

Or:

- 1 g/dose intravenously, with an additional dose of 1 g after 2 weeks;

### Treatments Under Evaluation

Studies have been carried out with ofatumumab, a second-generation anti-CD20-positive cell antibody, with potential use in cases of MN refractory to rituximab. There are limited data with the use of adrenocorticotropic hormone.

## MN Post-Kidney Transplantation

MN may appear in transplanted kidney as disease relapse in patients whose primary cause of chronic kidney disease was MN in the native kidney or as de novo glomerulopathy in patients who had another cause for their chronic kidney diseases.

### Relapsing MN

The reported incidence of MN in kidney transplant recipients with a previous history of this disease in native kidneys ranges from 5% to 44%^59,60,61,62^. This variation depends on the sample studied and the biopsy indications of each service. Relapses tend to occur early in the post-transplant period. In a study of 34 pre-transplant patients with MN, fifteen (44%) developed post-renal transplant relapse with a median of 13.6 months (range, 0.1 to 180.6 months)^63^. In that same report, two patterns of relapse were identified: early and late, and no predictors of relapses or progression were seen. In another study^61^, MN recurrence after transplantation occurred in 42% of patients, with a median of 4.0 months (range, 2 to 61 months). Patients with early relapse of MN, in both studies^61,63^, showed discrete or absent manifestations. On the contrary, nephrotic proteinuria was commonly found in patients with late relapses^61,63^. The relatively rapid relapse of MN after transplantation suggests the presence of a circulating factor at the time of transplantation, similar to the anti-PLA2R^16^ autoantibody, which has been reported in patients with relapsed MN^64–66^. Several studies have identified circulating anti-PLA2R antibodies at the time or after kidney transplantation as a risk factor for the development of recurrence^67,68,69,70^. On the other hand, the disappearance of circulating anti-PLA2R antibodies is associated with the improvement or resolution of proteinuria and its persistence is related to the worsening of the condition^69,71^. Therefore, monitoring circulating anti-PLA2R antibodies may have an impact on identifying patients who may benefit from increased maintenance immunosuppression or other types of medication^72,73^. Other target antigens have been associated in patients with MN relapse: THSD7A, NELL-1, EXT1/2, PCDH7 and Sema3B^74^.

The most common clinical manifestation is proteinuria, usually at non-nephrotic levels^63^. The association of circulating anti-PLA2R antibodies and its antigen in the renal tissue is also frequent in cases of recurrence^75^. However, some studies have not found this association with such evidence and there is a need to increase the number of patients investigated to prove whether in fact anti-PLA2R antibodies could predict the possibility of post-transplant relapse^67,69,71,76^.

Treatment of recurrent post-transplantation MN can be done conservatively, without increasing immunosuppression. If proteinuria is below 1 g/24 h measures such as inhibition of the renin-angiotensin system, strict control of blood pressure and hyperlipidemia should be implemented^77^. In cases of moderate to severe MN, with 24-hour proteinuria greater than 1g, the current suggestion is to administer rituximab as the drug of choice, although the most appropriate dose is still unknown^76^. Rituximab can cause partial or complete remission in most patients with relapse^76^. The administered doses of rituximab can vary from 1000 mg with an interval of one week, or 4 doses of 375 mg/m^2^/week, or other regimens reviewed in that same paper^76^. The authors advocate laboratory monitoring with CD19 B cell count, whose depletion occurs a few weeks after the administration of rituximab and/or blood levels of anti-PLA2R in MN associated with this antigen^76^. In this review^76^, the authors describe a total of 57 cases of MN recurrence in some references^63,67–70,78,79,80^, and there are reports of additional cases in other publications^81,82,83^. There are no other immunosuppressive therapies that have been shown to be more effective. There are attempts with bortezomib and other CD20 antibodies, such as obinutuzumab and ofatumumab, which have been described in isolated cases, especially in cases resistant to rituximab^76^. Recurrent MN can lead to graft loss and is more frequent, predominantly from 5 years after recurrences^59,78^.

### De Novo MN

The incidence of de novo MN is around 1.5 to 2%, but this incidence increases to up to 5.3% the longer the time after transplantation^84,85^. De novo MN may be even more prevalent in children with kidney transplants, reaching up to 9%^84^. De novo MN seems to be associated with chronic and/or antibody-mediated rejection, which can be shown in renal biopsy with classic MN findings and the presence of DSAs (donor specific antibodies), in patients with de novo MN^85,86,87^. The mechanisms linking the relationship between de novo MN and rejection is unknown, but some theories have been proposed focusing on the excess formation of circulating antigen-antibody complexes that are deposited on the glomerular basement membrane^87^. Glomerular injury caused by rejection facilitates the formation of subepithelial deposits.

Proteinuria due to de novo MN occurs many years after transplantation, usually after averages of 62.7 ± 44.4 months and 102.1 ± 68.3 months^87^. Many patients are asymptomatic and proteinuria generally remains in the subnephrotic range^85,87^. Diagnosis is made by findings on renal biopsy. To differentiate between relapse or de novo MN requires an accurate diagnosis of the original disease pre-kidney transplantation. If there is no way to define it, the assessment of anti-PLA2R associated with the diagnosis of post-transplant MN may be an alternative. De novo MN is not typically linked to the anti-PLA2R antibody, and in these cases the study results usually do not show the presence of this antibody^65,75^. Kidney biopsy in de novo MN may show findings consistent with rejection, evidence of transplant glomerulopathy such as positivity for CD4 in peritubular capillaries or duplication of the glomerular basement membrane, presence of DSAs, which may indicate an additional presence of chronic rejection mediated by antibodies^85,87^.

The natural history of de novo MN is unknown and is associated with renal graft loss in 50% of cases. It is not known whether this loss is due to the MN evolution or associated with other factors, such as chronic or active rejection mediated by antibodies^88,89^.

The most appropriate treatment for de novo MN has not been established. This is determined by the degree of proteinuria and whether or not renal function is stable. Conservative treatment as used in recurrent MN can also be applied considering patients with proteinuria less than 4.0 g/24h with stable renal function. Patients with proteinuria above this level and with worsening renal function are treated with rituximab, as previously described for recurrent MN. Other drugs such as cyclophosphamide have already been tested. Plasmapheresis can be considered when there are signs of rejection associated with MN.

## Conclusions

MN remains an important cause of nephrotic syndrome in adults. The remarkable discovery of PLA2R as a target antigen with the demonstration of its respective antibody circulating and deposited *in situ* in the glomerular subepithelial space has defined this disease as autoimmune, facilitating the monitoring of immunological activity and aiding in the decision to use immunosuppressants. The identification of several other target antigens (THSD7A, EXT1/2 and others) should contribute to advances in the knowledge of etiopathogenic mechanisms and provide better diagnostic classification and clinical evaluation. However, therapeutic options still result in only reasonable rates of clinical remission and high frequencies of adverse events.

Despite the many gaps that still exist in the knowledge of the mechanisms and treatment of this disease, we recognize that in the last 55 years there have been great advances in its understanding that were so well summarized in the title of a publication by Dr. William Couser^90^: “Membranous nephropathy: a long road but well-travelled”.
